# Emotional Exhaustion and Quiet Quitting Among Psychiatric Healthcare Workers: The Differential Mediating Roles of Emotional Labor Strategies

**DOI:** 10.3390/healthcare14203553

**Published:** 2026-10-21

**Authors:** Pan Yan, Hongmei Wang, Shuli Hu, Jie Li, Jiawei Tang, Hua Yu, Huaineng Wu

**Affiliations:** 1 Administration Department, Affiliated Mental Health Center & Hangzhou Seventh People’s Hospital, Zhejiang University School of Medicine, Hangzhou 310013, China; 2 Translational Psychiatry Research Laboratory, Affiliated Mental Health Center & Hangzhou Seventh People’s Hospital, Zhejiang University School of Medicine, Hangzhou 310013, China

**Keywords:** psychiatric healthcare workers, quiet quitting, emotional exhaustion, emotional labor, mediation analysis, network analysis

## Abstract

**Background/Objectives**: Psychiatric healthcare workers (HCWs) face unique emotional demands and may be particularly vulnerable to emotional exhaustion and quiet quitting. This study aimed to examine the association between emotional exhaustion and quiet quitting and the differential mediating roles of emotional labor strategies in this association. **Methods**: This multicenter cross-sectional study included 440 HCWs from three psychiatric hospitals in Hangzhou, China, between July and August 2025. Emotional exhaustion, emotional labor strategies, and quiet quitting were assessed using validated scales. Correlation, hierarchical regression, mediation, and network analyses were conducted. **Results**: Emotional exhaustion was positively associated with quiet quitting (β = 0.228, *p* < 0.001). Surface acting was positively correlated with emotional exhaustion and quiet quitting and partially mediated the association between them (indirect effect: β = 0.116, 95% CI: [0.071, 0.217]). Natural expression was negatively correlated with both emotional exhaustion and quiet quitting and also partially mediated their association (indirect effect: β = 0.056, 95% CI: [0.029, 0.115]). Deep acting was negatively correlated with quiet quitting but showed no significant indirect association. Network analysis identified lack of initiative, emotional exhaustion, and surface acting as central nodes, while emotional exhaustion, surface acting, and lack of motivation emerged as bridge nodes within the network. **Conclusions**: Emotional labor strategies showed distinct patterns in the association between emotional exhaustion and quiet quitting. Surface acting and natural expression showed significant indirect associations, whereas deep acting did not. These findings highlight the relevance of emotional exhaustion and strategy-specific emotional labor patterns in understanding quiet quitting among psychiatric HCWs.

## 1. Introduction

Quiet quitting has recently gained increasing attention as a workplace phenomenon [1]. It refers to a pattern in which employees perform the minimum required duties while avoiding extra-role responsibilities [1] and is characterized by work detachment, lack of initiative and lack of motivation [2]. Although quiet quitting may overlap conceptually with burnout and turnover intention, it represents a distinct construct. Unlike burnout, which is characterized by emotional exhaustion, depersonalization, and reduced personal accomplishment [3], quiet quitting primarily reflects a behavioral reduction in discretionary work effort. Unlike turnover intention, employees who engage in quiet quitting do not necessarily intend to leave their organizations; instead, they remain employed while limiting additional effort and organizational involvement [2,4].

In healthcare, the prevalence of quiet quitting is particularly concerning. Studies in Greece reported that 57.9% of healthcare workers (HCWs) engaged in quiet quitting [5], with 60.9% of nurses affected [6]. Quiet quitting has been associated with higher turnover intention and reduced organizational productivity, which may negatively impact patient care and healthcare outcomes [6,7]. Meta-analytic evidence shows a positive correlation between work-related stress and quiet quitting [8]. Psychiatric HCWs routinely manage patients with suicide risk, aggression, and severe emotional disturbances [9,10], requiring sustained emotional engagement and self-regulation during complex clinical interactions [11], and these demands may heighten their work-related stress and be associated with a greater tendency toward quiet quitting [8]. The potential consequences of quiet quitting may be particularly noteworthy in psychiatric care, as maintaining a stable and engaged workforce is important for the quality and continuity of mental health services [12]. Globally, mental health systems continue to face substantial workforce shortages [13]. Although quiet quitting does not necessarily involve leaving employment, reduced discretionary work effort and organizational involvement may place additional pressure on the effective capacity of already constrained mental health services. This challenge may be particularly relevant in China, where the psychiatric workforce remains insufficient, with only 3.64 psychiatrists per 100,000 people compared with 7.9 in high-income countries [14]. However, although quiet quitting has been examined among HCWs [8,15], evidence specific to psychiatric HCWs in China remains limited.

Emotional exhaustion, a key dimension of burnout, refers to a state of emotional depletion in which individuals feel overextended and drained of emotional resources [3]. Empirical evidence shows that emotional exhaustion is highly prevalent among HCWs [16] and is associated with adverse occupational outcomes, including job dissatisfaction [17] and turnover intention [18]. Among psychiatric HCWs, emotional exhaustion may be particularly salient given the sustained emotional demands, complex patient interactions, and occupational stress inherent in psychiatric care [11,19]. Although prior research has documented a positive association between emotional exhaustion and quiet quitting [20], the processes underlying this association, particularly among psychiatric HCWs, remain insufficiently understood.

Emotional labor refers to the process of managing feelings and emotional expressions in accordance with professional display expectations [21]. It is commonly operationalized through three strategies: surface acting, natural expression, and deep acting [22]. Surface acting involves modifying emotional expressions without changing internal feelings [21,22]. Natural expression, by contrast, involves the spontaneous expression of naturally felt emotions that align with organizational expectations [22]. Deep acting involves adjusting internal feelings to genuinely experience the required emotions [21,22]. Psychiatric HCWs face high emotional labor demands [11,23]. Their work frequently requires them to regulate their own emotional responses while simultaneously managing patients with substantial emotional and behavioral difficulties [10,24]. Such emotional regulation may be particularly challenging in the demanding and complex clinical encounters characteristic of psychiatric care [10,24]. Importantly, these emotional labor strategies differ in how employees regulate their emotions and may therefore have different implications for emotional resources and work-related behaviors.

The Conservation of Resources (COR) theory posits that individuals are motivated to acquire, preserve, and protect valued resources [25]. It also emphasizes the primacy of resource loss and resource loss spirals, whereby individuals with fewer resources become more vulnerable to further losses [26]. When employees experience sustained emotional exhaustion, they may have fewer emotional resources available and may seek to protect their remaining resources by reducing discretionary work investment [25,26]. For psychiatric HCWs, quiet quitting may therefore represent a resource-conserving response to emotional exhaustion. In this context, emotionally exhausted HCWs may remain employed while limiting extra-role effort, initiative, and psychological involvement rather than leaving the organization.

Emotional labor strategies may further help explain the association between emotional exhaustion and quiet quitting because different strategies involve distinct patterns of resource expenditure, conservation, and acquisition. From a COR perspective, emotionally exhausted HCWs may be more likely to rely on surface acting to meet emotional display requirements without changing their underlying feelings. Supporting this proposed direction, longitudinal evidence from a four-wave cross-lagged study of newly hired nurses showed that emotional exhaustion positively predicted subsequent surface acting, whereas surface acting did not predict subsequent emotional exhaustion [27]. Although whether the same temporal pattern applies to psychiatric HCWs remains to be tested, this finding provides preliminary support for the proposed direction from emotional exhaustion to surface acting. The discrepancy between felt and displayed emotions inherent in surface acting requires sustained self-regulation and may be associated with further resource depletion [28,29]. Persistent emotional dissonance may also be associated with less favorable interpersonal interactions, potentially limiting opportunities for resource replenishment [30]. As resources become increasingly strained, greater reliance on surface acting may therefore be associated with a stronger tendency to reduce discretionary work effort, consistent with quiet quitting.

Natural expression may involve a different resource process. Because naturally felt emotions are aligned with professional display expectations, natural expression involves less emotional dissonance and may require fewer regulatory resources [22]. From a COR perspective, these lower regulatory demands may help conserve emotional resources, while authentic emotional expression may facilitate interpersonal interactions [31] that provide opportunities for social and emotional resource acquisition. Nevertheless, natural expression should not be regarded as entirely effortless in psychiatric care. Expressing naturally felt emotions in ways that remain responsive to professional and patient-care demands may still require emotional resources. Emotionally exhausted HCWs may therefore find it more difficult to sustain natural emotional expression that remains aligned with professional expectations. In turn, lower natural expression may limit opportunities for authentic interpersonal connection and resource replenishment, and may therefore be associated with a stronger tendency toward quiet quitting.

From a COR perspective, deep acting can be understood as a form of resource investment [26,32]. Although modifying internal feelings to align them with expected emotional displays requires cognitive and emotional resources, the resulting congruence between felt and displayed emotions may reduce emotional dissonance and facilitate authentic interpersonal interactions [21,29,32]. Such interactions may provide opportunities for social and emotional resource acquisition [32]. Emotionally exhausted HCWs, however, may have fewer emotional and cognitive resources available to engage consistently in this effortful regulatory strategy. In turn, lower reliance on deep acting may be associated with fewer opportunities for resource acquisition and a stronger tendency toward quiet quitting.

However, despite growing research on emotional exhaustion, emotional labor strategies, and quiet quitting, to our knowledge, no study has integrated these constructs within a single analytical framework among psychiatric HCWs. In particular, it remains unclear whether surface acting, natural expression, and deep acting play distinct mediating roles in the association between emotional exhaustion and quiet quitting in this population.

To address these gaps and provide a more differentiated understanding of this association, we draw on COR theory and prior research to advance the following hypotheses:

**H1.**  *Emotional exhaustion is positively associated with quiet quitting.*

**H2.**  *Surface acting is positively associated with both emotional exhaustion and quiet quitting, and mediates the relationship between emotional exhaustion and quiet quitting.*

**H3.**  *Natural expression is negatively associated with both emotional exhaustion and quiet quitting, and mediates the relationship between emotional exhaustion and quiet quitting.*

**H4.**  *Deep acting is negatively associated with both emotional exhaustion and quiet quitting, and mediates the relationship between emotional exhaustion and quiet quitting.*

In addition to the hypothesized mediation model, this study uses network analysis to provide a complementary perspective. While the mediation model examines associations among emotional exhaustion, emotional labor strategies, and quiet quitting at the construct level, network analysis explores the interconnections among their individual indicators. This approach can identify central nodes within the network and bridge nodes connecting different communities.

Accordingly, this study aims to: (1) examine the association between emotional exhaustion and quiet quitting among psychiatric HCWs; (2) examine the mediating roles of surface acting, natural expression, and deep acting in the association between emotional exhaustion and quiet quitting; and (3) explore the network structure and identify central and bridge nodes among emotional exhaustion, emotional labor strategies, and quiet quitting.

## 2. Materials and Methods

### 2.1. Study Design

This multicenter cross-sectional study was conducted in accordance with the STROBE guidelines.

### 2.2. Setting and Sample

Participants were recruited between July and August 2025 from three psychiatric hospitals at different administrative levels in Hangzhou, China, using convenience sampling. All psychiatrists and nurses who were directly involved in patient care in psychiatric departments at the three participating hospitals and had at least one year of clinical practice experience were invited to participate on a voluntary basis. Exclusion criteria were: (1) individuals with severe physical or mental health conditions; (2) staff on temporary duty, rotation, or internship; and (3) visiting or trainee personnel.

An online questionnaire was distributed to the 496 eligible participants via DingTalk. A total of 459 completed questionnaires were returned. Of these, 19 questionnaires were excluded due to patterned responses (*n* = 11), failed attention checks (*n* = 5), or implausible responses (*n* = 3). Three attention-check items were embedded throughout the questionnaire, and responses were considered invalid if participants failed any of these checks. The final analytic sample comprised 440 participants, yielding a valid response rate of 88.71%.

To verify that this sample size was adequate for the planned mediation analyses, a power analysis was conducted using the Monte Carlo approach recommended by Schoemann et al. [33]. Assuming a model with three parallel mediators, moderate bivariate correlations (r = 0.30) between the independent variable, mediators, and dependent variable, and low inter-mediator correlations (r = 0.20), the analysis indicated that a minimum of 333 participants was required to achieve 95% power at the 95% confidence level (5000 replications, 20,000 Monte Carlo draws per replication). The final sample of 440 exceeded this minimum. No missing data were present for any study variable because the questionnaire required completion of all items before submission.

### 2.3. Measurements

#### 2.3.1. Demographic Characteristics

Demographic characteristics collected included gender, age, employment type, professional title, education level, monthly night shifts, psychiatric healthcare working experience, and profession.

#### 2.3.2. Emotional Exhaustion

Emotional exhaustion among psychiatric HCWs was measured using the emotional exhaustion subscale of the Maslach Burnout Inventory-General Survey [34]. The 5-item subscale was rated on a 5-point Likert scale ranging from 1 (“strongly disagree”) to 5 (“strongly agree”), consistent with previous studies among Chinese healthcare professionals [18,35]. Total scores were calculated by summing all item scores, with higher scores indicating greater emotional exhaustion. A representative item is “I feel emotionally drained from my work.” The scale demonstrated excellent reliability in our sample, with a Cronbach’s α of 0.922.

#### 2.3.3. Emotional Labor

Emotional labor was measured using the Emotional Labor Scale developed by Diefendorff et al. [22]. This 14-item scale consists of three dimensions: surface acting (7 items), natural expression (3 items), and deep acting (4 items). Items were rated on a 5-point Likert scale ranging from 1 (“strongly disagree”) to 5 (“strongly agree”). Subscale scores were calculated by summing the corresponding item scores, with higher scores indicating greater use of each emotional labor strategy. Representative items include surface acting (“I just pretend to have the emotions I need to display for my job”), natural expression (“The emotions I express to others come naturally”), and deep acting (“I make an effort to actually feel the emotions that I need to display toward others”). The scale has demonstrated good reliability in Chinese populations [36]. In our study, Cronbach’s α was 0.901 for surface acting, 0.825 for natural expression, and 0.701 for deep acting.

#### 2.3.4. Quiet Quitting

Quiet quitting among psychiatric HCWs was measured using the Quiet Quitting Scale, which was originally developed by Galanis et al. [2] and later validated in Chinese populations by Zhang et al. [37]. The 9-item scale comprises three factors: work detachment (4 items), lack of initiative (3 items), and lack of motivation (2 items). Responses were rated on a 5-point Likert scale ranging from 1 (“strongly disagree”) to 5 (“strongly agree”), with items 7–9 reverse-scored. Total scores were calculated by summing all item scores, with higher scores indicating greater quiet quitting tendencies. Individuals with an average item score of 2.06 or above were categorized as quiet quitters [38]. A representative item is: “I only do the basic or minimum required work, and I won’t do anything above and extra.” The scale exhibited adequate internal consistency, with a Cronbach’s α of 0.794.

### 2.4. Ethical Considerations

The study was conducted in accordance with the Declaration of Helsinki and approved by the Ethics Committee of Hangzhou Seventh People’s Hospital (protocol code: 2024-074). Prior to participation, all participants were informed about the study purpose, its voluntary and anonymous nature, and the confidentiality of their responses. They were informed that no personally identifiable information would be collected, that raw data would be accessible only to the research team, and that aggregated results would not be shared with hospital administrators or used for performance evaluation or personnel decisions. Electronic informed consent was obtained from all participants before they proceeded to the questionnaire.

### 2.5. Statistical Analysis

Statistical analyses were performed using SPSS (version 27.0, IBM Corp., Armonk, NY, USA) and R (version 4.4.3, R Foundation for Statistical Computing, Vienna, Austria). Prior to the main analyses, the assumptions for each statistical test were assessed (e.g., normality, linearity, homoscedasticity, multicollinearity). All assumptions were satisfied, except for homogeneity of variances for the gender comparison. Potential common method bias was assessed using Harman’s single-factor test together with the procedural measures implemented during data collection.

Demographic differences in quiet quitting were assessed using independent-samples *t*-tests (Welch’s *t*-test for gender, Student’s *t*-test for other binary variables) or one-way analysis of variance (ANOVA). Where ANOVA yielded significant results, post hoc pairwise comparisons were performed using the Bonferroni correction. Relationships among the scale scores were assessed using Pearson correlation analysis.

Hierarchical multiple linear regression was applied to examine the effects of demographic characteristics, emotional exhaustion, and emotional labor on quiet quitting. Mediation analysis was subsequently conducted using PROCESS Model 4, with indirect effects tested using 5000 bootstrap resamples and considered statistically significant when the 95% confidence intervals (CIs) did not include zero.

A Gaussian graphical model was estimated using the EBICglasso algorithm. Expected influence (EI) was calculated to identify central nodes, and bridge expected influence (BEI; one-step) was computed to identify bridge nodes connecting different communities. Edge-weight accuracy was assessed using nonparametric bootstrapping with 1000 samples to derive 95% CIs. The stability of centrality indices was evaluated using case-dropping bootstrapping with 1000 samples, yielding correlation stability (CS) coefficients; values above 0.50 were interpreted as indicating good stability.

## 3. Results

### 3.1. Common Method Bias Test

Harman’s single-factor test showed that the first factor accounted for 30.54% of the total variance, below the 40% threshold, indicating that common method bias was unlikely to have substantially influenced the findings.

### 3.2. Participant Characteristics

The final sample comprised 440 HCWs, with a mean age of 38.35 ± 8.32 years. The majority were female (82.50%), had junior or intermediate professional titles (75.68%) and held bachelor’s degrees (80.23%). Most participants were permanent employees (83.18%); 39.55% reported more than 5 night shifts per month, and 40.45% had 11–20 years of psychiatric healthcare working experience. By profession, 72.05% were nurses and 27.95% were psychiatrists. Significant differences in quiet quitting scores were observed across gender, professional title, monthly night shifts, working experience, and employment type (Table 1).

### 3.3. Correlation Analysis

In this study, 62.50% of HCWs met the proposed cutoff for quiet quitting, with a mean score of 20.03 ± 4.85. Pearson correlations among the study variables are presented in Table 2. Emotional exhaustion was positively correlated with quiet quitting (r = 0.421, *p* < 0.01). Surface acting was positively correlated with emotional exhaustion (r = 0.538, *p* < 0.01) and quiet quitting (r = 0.458, *p* < 0.01). In contrast, natural expression was negatively correlated with emotional exhaustion (r = −0.273, *p* < 0.01) and quiet quitting (r = −0.407, *p* < 0.01). Deep acting showed a negative correlation with quiet quitting (r = −0.212, *p* < 0.01), but no significant correlation with emotional exhaustion (r = −0.024, *p* = 0.612).

### 3.4. Hierarchical Multiple Linear Regression Analysis

As shown in Table 3, hierarchical multiple linear regression analysis was conducted to examine the contributions of demographic characteristics, emotional exhaustion, and emotional labor strategies to quiet quitting. In the first step, demographic variables explained 7.4% of the variance in quiet quitting. Specifically, being female (β = −0.169, *p* < 0.001) and longer working experience (11–20 years vs. ≤10 years, β = −0.140, *p* = 0.003) were associated with lower levels of quiet quitting, whereas higher frequency of monthly night shifts (>5 vs. 0, β = 0.183, *p* < 0.001) was associated with higher levels of quiet quitting. The inclusion of emotional exhaustion significantly increased the model’s explanatory power (ΔR^2^ = 0.159, *p* < 0.001), bringing the total R^2^ to 23.3%. Moreover, emotional exhaustion was positively associated with quiet quitting (β = 0.401, *p* < 0.001). With the further incorporation of emotional labor strategies, the model’s explanatory power increased to 34.9%. Surface acting was positively associated with quiet quitting (β = 0.277, *p* < 0.001), while both natural expression and deep acting were negatively associated with quiet quitting (β = −0.107, *p* = 0.042; β = −0.174, *p* < 0.001). Emotional exhaustion remained positively associated with quiet quitting in the final model (β = 0.223, *p* < 0.001).

### 3.5. Mediation Analysis of Emotional Labor Strategies on the Relationship Between Emotional Exhaustion and Quiet Quitting

According to the findings of Pearson correlation and hierarchical multiple linear regression analyses, we selected surface acting and natural expression as candidate mediators for the subsequent mediation analysis, considering that these two dimensions of emotional labor were related to both emotional exhaustion and quiet quitting, while gender, monthly night shifts, and working experience were included as covariates.

**Figure 1:**
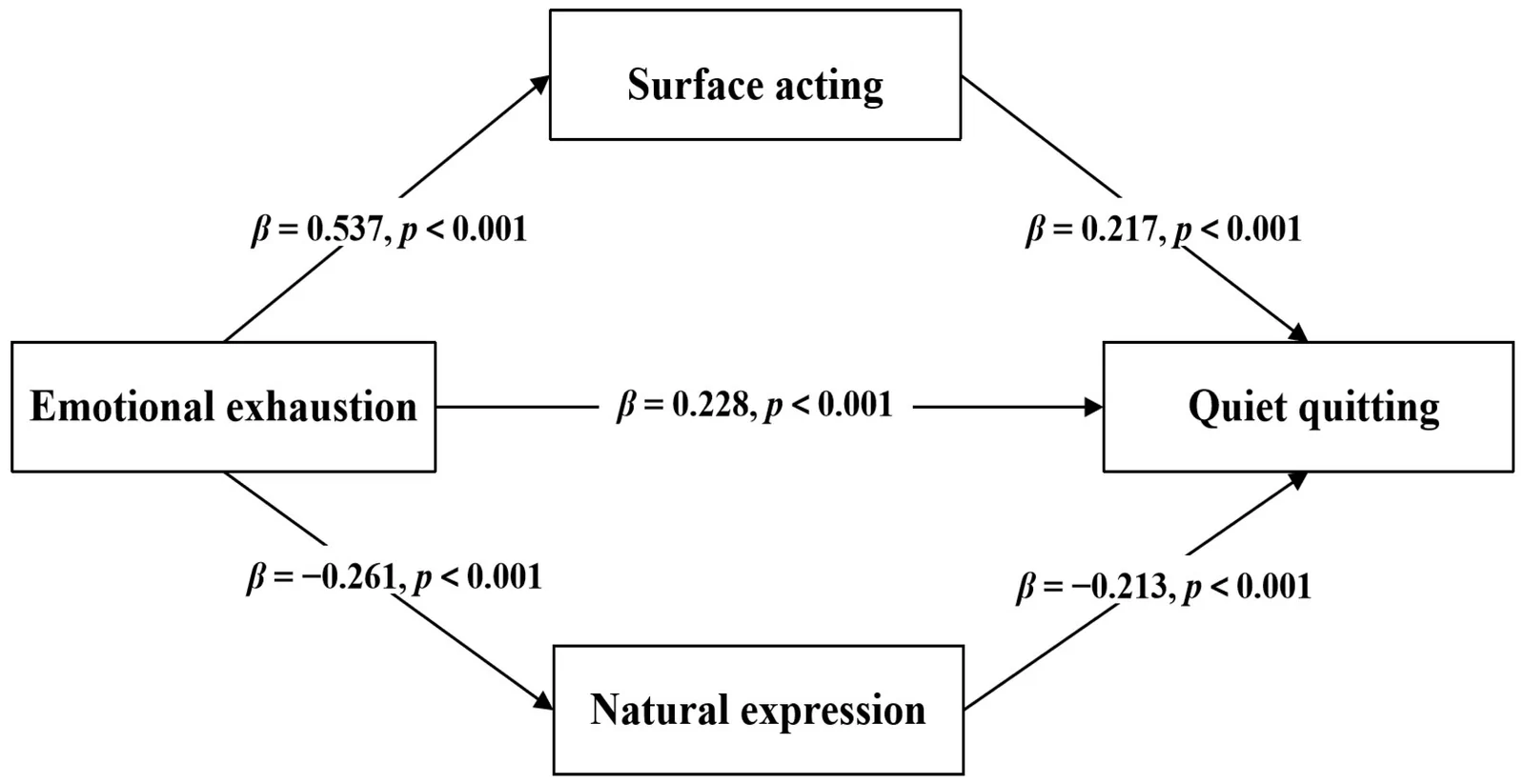
The mediating roles of emotional labor strategies in the association between emotional exhaustion and quiet quitting.

As shown in Table 4 and Figure 1, emotional exhaustion was positively associated with quiet quitting (total effect: β = 0.400, *p* < 0.001; direct effect: β = 0.228, *p* < 0.001). Emotional exhaustion was also positively associated with surface acting (β = 0.537, *p* < 0.001) and negatively associated with natural expression (β = −0.261, *p* < 0.001). Surface acting was positively associated with quiet quitting (β = 0.217, *p* < 0.001), and partially mediated the association between emotional exhaustion and quiet quitting (indirect effect: β = 0.116, 95% CI [0.071, 0.217]), accounting for 29.0% of the total effect. Natural expression was negatively associated with quiet quitting (β = −0.213, *p* < 0.001), and partially mediated the association (indirect effect: β = 0.056, 95% CI [0.029, 0.115]), accounting for 14.0% of the total effect.

### 3.6. Network Analysis

**Figure 2:**
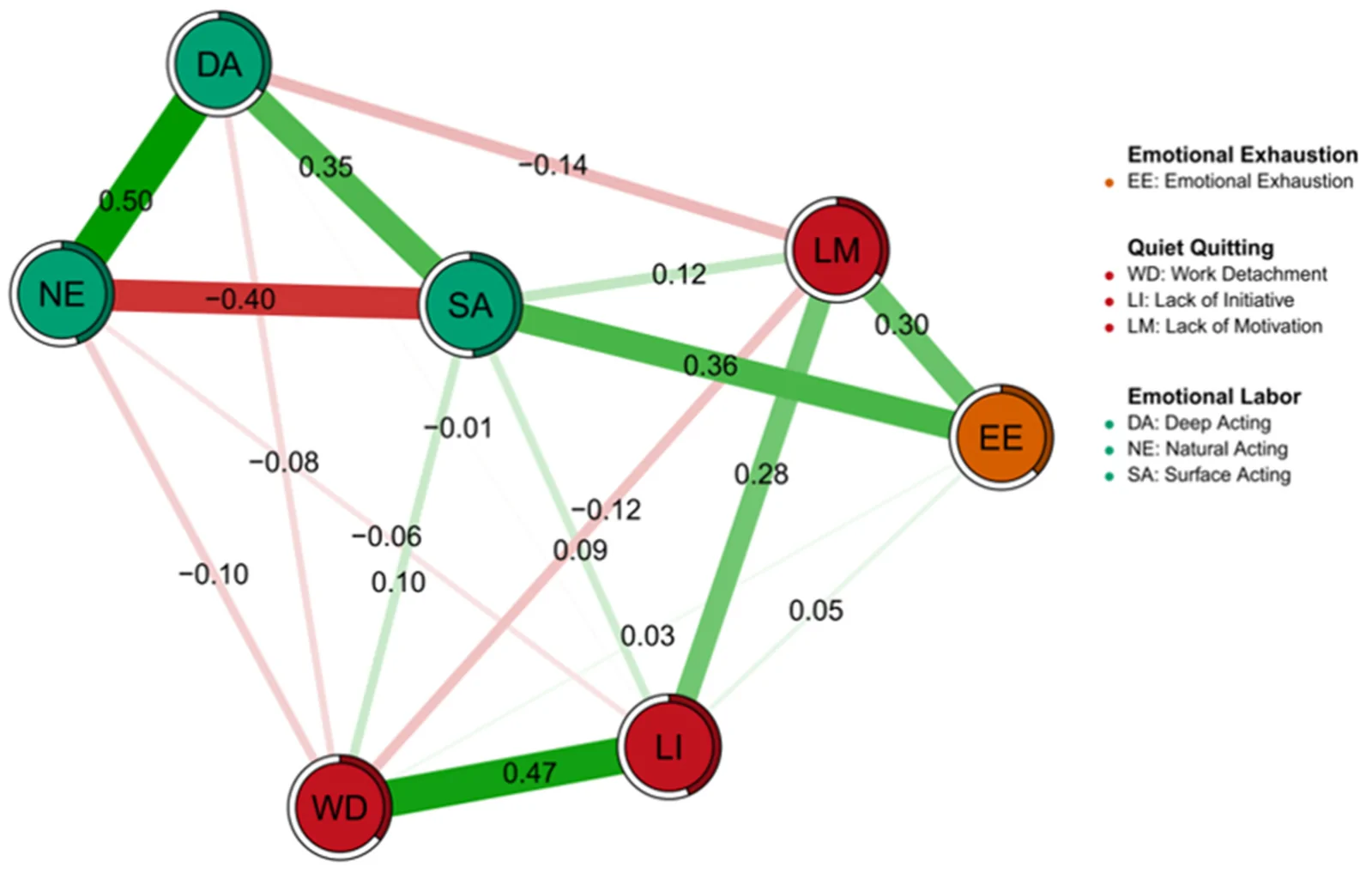
The network of emotional exhaustion, emotional labor strategies, and quiet quitting. Green edges represent positive associations, whereas red edges represent negative associations. Thicker edges between nodes indicate stronger associations.

The network analysis examined the connections among emotional exhaustion, emotional labor strategies (surface acting, natural expression, and deep acting), and quiet quitting (work detachment, lack of initiative, lack of motivation). The estimated network comprised 7 nodes and 18 nonzero edges, with a mean edge weight of 0.198. Within the emotional labor community, deep acting was positively associated with surface acting and natural expression, whereas surface acting was negatively associated with natural expression. Within the quiet quitting community, lack of initiative was positively linked to work detachment and lack of motivation, whereas work detachment showed a negative association with lack of motivation. Edges spanning communities indicated that emotional exhaustion was positively linked to surface acting, lack of motivation, and lack of initiative (Figure 2).

**Figure 3:**
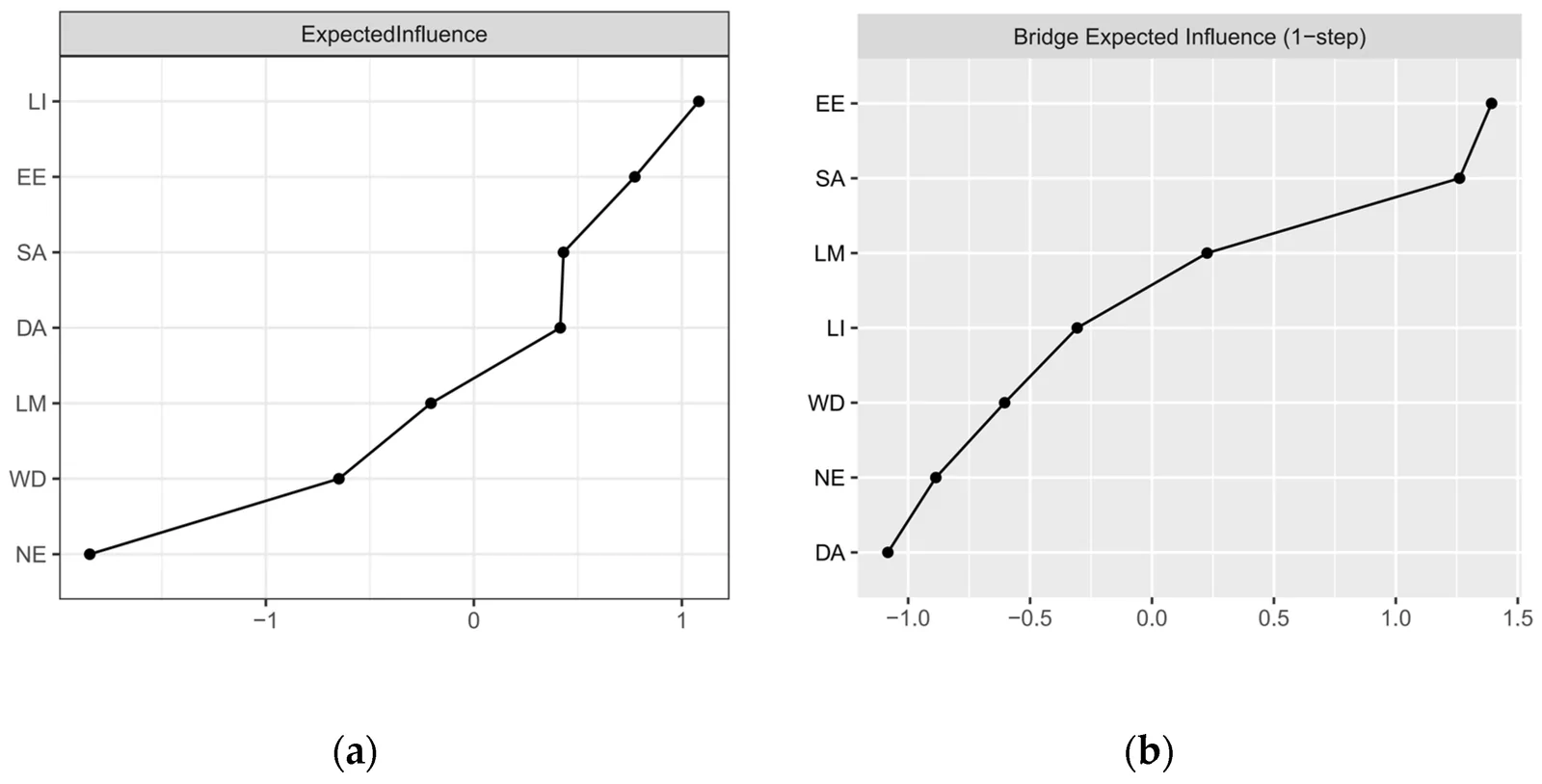
Centrality indices of the estimated network. (**a**) Expected Influence. (**b**) Bridge Expected Influence. EE, emotional exhaustion; SA, surface acting; NE, natural expression; DA, deep acting; WD, work detachment; LI, lack of initiative; LM, lack of motivation.

As shown in Figure 3a, lack of initiative, emotional exhaustion, and surface acting emerged as the most central nodes, exhibiting the highest EI. Figure 3b further identified emotional exhaustion, surface acting, and lack of motivation as bridge nodes with the highest BEI.

**Figure 4:**
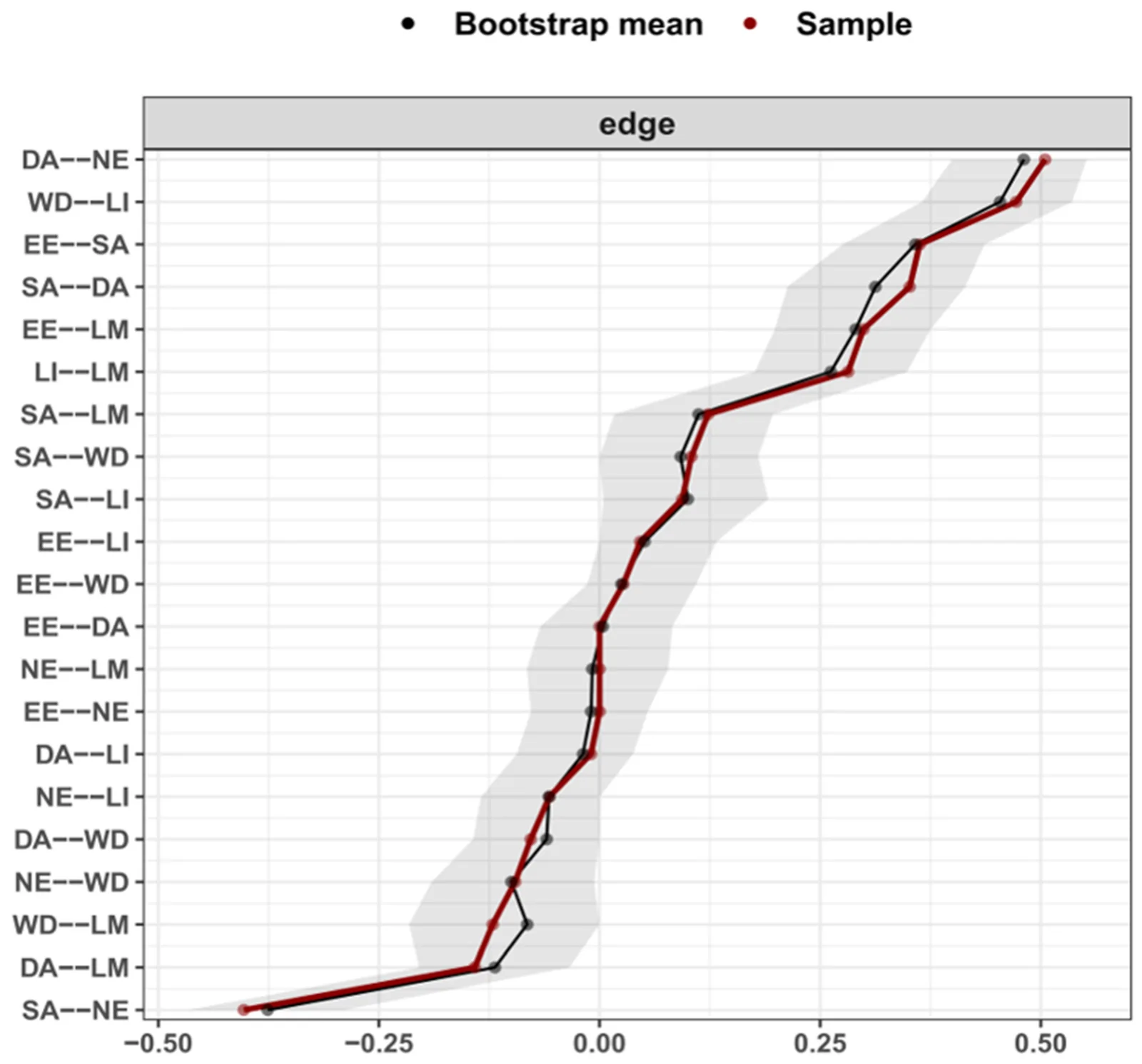
Accuracy of edge weights. EE, emotional exhaustion; SA, surface acting; NE, natural expression; DA, deep acting; WD, work detachment; LI, lack of initiative; LM, lack of motivation.

**Figure 5:**
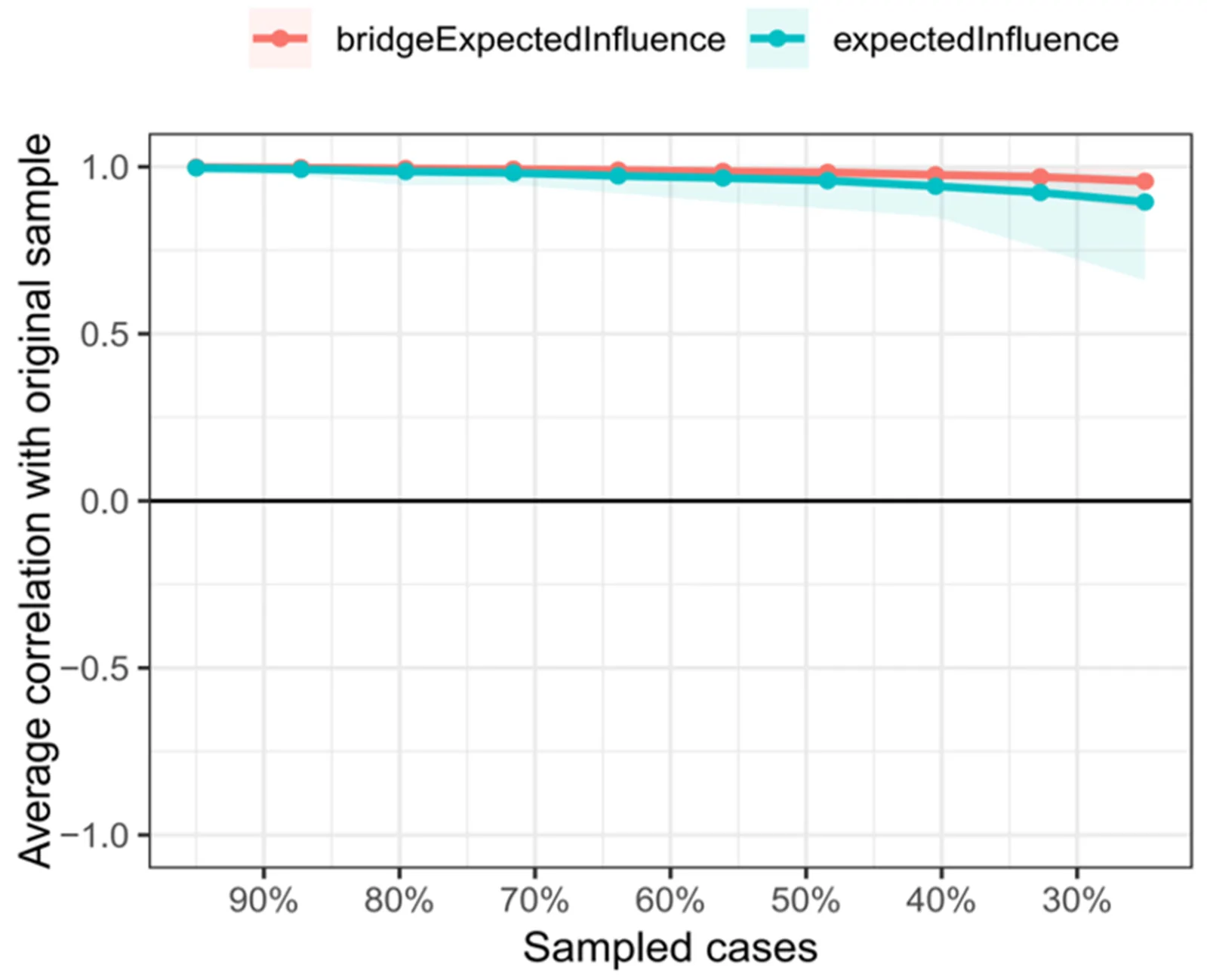
Centrality stability assessed via case-dropping bootstrapping.

The accuracy of edge weights was assessed using nonparametric bootstrapping. As shown in Figure 4, the 95% CIs were narrow, indicating acceptable accuracy. Centrality stability was further examined via case-dropping bootstrapping (Figure 5). The resulting CS coefficients were 0.75 for both EI and BEI, indicating good stability.

## 4. Discussion

This study examined the association between emotional exhaustion and quiet quitting among psychiatric HCWs in China and explored the mediating roles of distinct emotional labor strategies in this association. Emotional exhaustion was positively associated with quiet quitting. Surface acting and natural expression showed significant indirect associations, whereas deep acting did not. Additionally, integrating mediation analysis with network analysis provided complementary perspectives on the associations among emotional exhaustion, emotional labor strategies, and quiet quitting at the construct level and on the interconnections among their individual indicators.

In the present study, 62.50% of participants met the proposed cutoff for quiet quitting. Several contextual features of psychiatric care may be relevant to this finding. Psychiatric HCWs may face substantial emotional labor demands due to frequent interactions with patients experiencing severe psychological distress [11,24]. Exposure to workplace violence and unpredictable patient behavior may add further occupational challenges [10,39], while stigma surrounding mental illness may create additional social and professional pressures for psychiatric HCWs [40]. Although these factors were not directly examined in the present study, they may help contextualize the observed prevalence of quiet quitting and warrant further investigation.

Regarding demographic characteristics, being male, shorter work experience, and frequent night shifts were associated with higher quiet quitting tendencies. The association between male gender and quiet quitting may also be understood in the context of traditional gender-role expectations in Chinese culture. Such expectations may place greater emphasis on men’s breadwinner responsibilities [41], potentially increasing work-related pressure, as well as on self-reliance, which may discourage help-seeking for psychological distress [42]. Among less experienced psychiatric HCWs, challenges related to early-career professional adaptation and organizational commitment may be relevant to their higher quiet quitting tendencies [43,44]. Frequent night shifts may involve additional occupational strain related to circadian rhythm disruption [45] and work–life conflict [46], which may be relevant to the observed association with quiet quitting. Importantly, these findings may help identify psychiatric HCWs with higher quiet quitting tendencies, highlighting the need for tailored support for these groups.

Emotional exhaustion was positively associated with quiet quitting among psychiatric HCWs, thereby supporting H1. Drawing on COR theory, sustained work stress may contribute to resource depletion, which may be reflected in emotional exhaustion [26]. When the work environment does not adequately replenish these depleted resources, exhaustion may intensify, and HCWs may exhibit quiet quitting as a potential resource-conservation response characterized by deliberately limiting effort to prevent further resource loss [26,47]. This interpretation aligns with previous research linking burnout, of which emotional exhaustion is a key component, to higher quiet quitting tendencies [4,48], and is further supported by a recent comprehensive scoping review [1]. Collectively, these findings highlight emotional exhaustion as an important correlate of quiet quitting among psychiatric HCWs and underscore the potential value of early identification and timely support.

Surface acting was positively associated with both emotional exhaustion and quiet quitting and partially mediated the relationship between emotional exhaustion and quiet quitting, thus supporting H2. Previous studies have also reported associations between emotional exhaustion and surface acting among HCWs [27,49]. According to COR theory, emotional exhaustion may be associated with greater reliance on surface acting, as individuals experiencing resource depletion may attempt to manage ongoing job demands while conserving remaining psychological resources [25,29]. This pattern may also be understood in light of the resource loss spirals proposed by COR theory, whereby initial resource depletion may increase vulnerability to further resource loss [26]. For emotionally exhausted psychiatric HCWs, surface acting may represent a more readily adopted strategy for managing ongoing emotional demands [27]. This pattern may be particularly relevant to psychiatric HCWs, whose work often involves sustained compassionate interactions with patients [11]; the dissonance between internal feelings and emotional expressions may undermine emotional authenticity and be associated with further resource depletion [24]. Within this theoretical framework, greater resource depletion may be associated with reduced work investment and greater psychological distance from work, which is consistent with the behavioral profile of quiet quitting [26,47].

In contrast, natural expression was negatively associated with emotional exhaustion and quiet quitting and partially mediated the relationship between emotional exhaustion and quiet quitting, thus supporting H3. Previous research has shown that natural expression among nurses is associated with lower emotional exhaustion and professional inefficacy [50], as well as better nurse-patient relationships and greater job satisfaction [31]. From the perspective of COR theory, this finding may be interpreted in relation to the relatively low emotional dissonance and potential resource-preserving role of natural expression [22,51]. Specifically, emotionally exhausted HCWs may have fewer psychological resources to sustain naturally felt emotional expressions that remain aligned with professional expectations during demanding patient interactions [22,26]. Lower natural expression may therefore reflect a reduced capacity for authentic interpersonal engagement, which may be associated with fewer positive relational experiences and weaker discretionary work investment [31]. In this sense, the negative association between natural expression and quiet quitting suggests that authentic emotional expression may be relevant to understanding lower quiet quitting tendencies among psychiatric HCWs.

Despite its negative association with quiet quitting, deep acting was not associated with emotional exhaustion and the indirect association through deep acting was not significant, thereby only partially supporting H4. Previous studies have reported mixed findings. Some studies reported a negative association, suggesting that deep acting may be associated with lower emotional exhaustion [27,52,53], while others found no significant relationship [24,54], which aligns with our findings. This null finding may be explained by several factors. One possible explanation is the heterogeneity of deep acting itself. Deep acting may encompass two distinct dimensions: engagement-focused regulation and disengagement-focused regulation [55]. These strategies may show opposing associations with emotional exhaustion. Another possible explanation relates to the emotional demands of psychiatric care. Psychiatric HCWs frequently encounter challenging patient interactions and complex emotional demands, making emotional labor an important component of psychiatric healthcare practice [10,11]. Such complex interactions may require HCWs to continuously monitor patients’ emotional and behavioral states and respond to clinical risks, such as suicide or aggression, while simultaneously regulating their own emotional responses and maintaining appropriate therapeutic interactions [9,10,24]. In this context, deep acting may require sustained cognitive and emotional effort to regulate internal feelings and maintain professional emotional displays. Although deep acting is generally considered a more adaptive emotional regulation strategy than surface acting, its potential benefits may be offset by the substantial cognitive and emotional resources required to manage the intensity and unpredictability of interactions with psychiatric patients. Previous studies have shown that deep acting is associated with favorable work outcomes, such as job engagement and job satisfaction [56,57]. Consistent with these findings, the negative association between deep acting and quiet quitting observed in our study may reflect potential resource-related benefits of this strategy. Although deep acting requires cognitive and emotional resource investment, greater congruence between felt and displayed emotions may reduce emotional dissonance and facilitate more authentic interpersonal interactions [21,29]. These potential benefits may help explain why deep acting was associated with lower quiet quitting tendencies even though its association with emotional exhaustion was not significant.

Furthermore, network analysis provided an indicator-level perspective that complemented the construct-level findings on indirect associations. We found that emotional exhaustion occupied one of the most central positions in the network, exhibiting the highest BEI and the second-highest EI. These findings suggest that emotional exhaustion may represent an important node connecting different communities within the network structure. Its strong positive edge with surface acting indicated a close association between emotional exhaustion and surface acting, which is theoretically consistent with the resource conservation perspective [32]. Yet this inauthentic display may be associated with further resource loss and may represent a connection between emotional exhaustion and the quiet quitting community. Lack of motivation showed strong cross-community edges, serving as a structural bridge linking the quiet quitting community to emotional exhaustion and surface acting. This pattern aligns with Huang et al. [23], who identified surface acting as the principal bridge linking emotional labor to burnout in psychiatric nurses. The present study extends this line of inquiry by applying a network bridge perspective to quiet quitting and suggests that lack of motivation may serve as the cross-community bridge, whereas lack of initiative may represent the core feature of quiet quitting. Collectively, these findings suggest that emotional exhaustion, surface acting, and lack of motivation may represent structurally important correlates within the observed network. However, given the exploratory and cross-sectional nature of the network analysis, these nodes should be interpreted as hypothesis-generating indicators rather than confirmed intervention targets.

### 4.1. Limitations and Future Research

Although this study provides valuable insights, several limitations should be acknowledged. First, the cross-sectional design limits causal interpretation and prevents conclusions about the temporal direction of the association between emotional exhaustion and quiet quitting. Second, the reliance on self-reported data may increase the risk of social desirability bias and common method bias. Although Harman’s single-factor test did not indicate substantial common method bias, the possibility of such bias cannot be completely excluded. Third, although the final sample size exceeded the minimum sample size estimated by the Monte Carlo power analysis, participants were recruited through convenience sampling from only three psychiatric hospitals in a single city in China, which may introduce selection bias and limit sample representativeness, thereby restricting the generalizability of the findings to other regions, healthcare settings, and cultural contexts. Fourth, although the mediation models adjusted for several demographic characteristics, important occupational and contextual factors, such as workplace violence, perceived organizational support, and stigma, were not measured; thus, residual confounding cannot be ruled out. Future research should adopt longitudinal designs, including diary-based or experience-sampling approaches, to further clarify the temporal relationships among emotional exhaustion, emotional labor strategies, and quiet quitting. Studies should also incorporate objective indicators and multi-source data, such as supervisor evaluations, to reduce potential biases associated with self-reported data, and consider relevant occupational and contextual factors to better account for potential confounding. In addition, studies that use sampling strategies such as stratified sampling and recruit larger and more geographically diverse samples may help enhance sample representativeness and strengthen the generalizability of the findings across different cultural and healthcare settings.

### 4.2. Theoretical and Practical Implications

Theoretically, this study contributes to the integration of COR theory, emotional labor theory, and the quiet quitting literature to better understand quiet quitting among psychiatric HCWs. Rather than conceptualizing the association between emotional exhaustion and quiet quitting as purely direct, the findings suggest that the association may involve strategy-specific emotional labor patterns. Surface acting and natural expression showed significant indirect associations, whereas deep acting was negatively associated with quiet quitting but showed no significant indirect association. These findings support a differentiated understanding of emotional labor in psychiatric healthcare, where emotional regulation is embedded in patient-care demands. The integration of network analysis further extends this interpretation by providing an indicator-level perspective complementary to the construct-level mediation model.

From a practical perspective, these findings may inform several tentative directions for organizational support and future intervention development. Healthcare institutions may consider monitoring emotional exhaustion and identifying work conditions associated with sustained emotional depletion, especially for HCWs on frequent night shifts or high-demand work arrangements. Supportive workplace practices, such as workload review, peer support, and access to psychological support, may be relevant for addressing emotional strain. Given the higher quiet quitting scores observed among nonpermanent staff, tailored organizational support could focus on ensuring equitable access to professional development opportunities, supervisory guidance, clear role communication, and workplace resources. In addition, training programs aimed at helping HCWs recognize and manage emotional labor demands, reduce excessive reliance on surface acting, and support more authentic emotional expression could also be explored. At the policy level, these findings may inform the development of policies and programs to promote psychiatric workforce stability by drawing attention to quiet quitting and its associations with emotional exhaustion and emotional labor, particularly in the context of global mental health workforce shortages.

## 5. Conclusions

This study provides preliminary evidence that emotional exhaustion is associated with quiet quitting among psychiatric HCWs and that different emotional labor strategies may play distinct roles in this association. Specifically, surface acting and natural expression showed significant but divergent indirect associations, whereas deep acting was associated with lower quiet quitting tendencies but did not show a significant indirect association. From a COR perspective, these findings suggest that different emotional labor strategies may reflect distinct patterns of psychological resource regulation among psychiatric HCWs under conditions of emotional exhaustion. Network analysis further suggested that lack of initiative, emotional exhaustion, and surface acting were central nodes, while emotional exhaustion, surface acting, and lack of motivation were identified as important bridge nodes within the network. Overall, these findings highlight the importance of considering emotional exhaustion and strategy-specific emotional labor patterns in understanding quiet quitting among psychiatric HCWs.

## Figures and Tables

**Table 1:** Demographic characteristics of the participants.

Characteristics	*n* (%)	Quiet Quitting		
		Mean ± SD	F/t	*p*
Gender ^b^				
Male	77 (17.50)	21.65 ± 6.00	2.712	0.008
Female	363 (82.50)	19.69 ± 4.51		
Professional title ^c^				
Junior and below	141 (32.04)	20.87 ± 4.94	3.353	0.036 ^d^
Intermediate	192 (43.64)	19.49 ± 5.02		
Senior	107 (24.32)	19.91 ± 4.29		
Education level ^c^				
Junior college	21 (4.77)	19.62 ± 4.78	0.210	0.810
Bachelor’s degree	353 (80.23)	20.00 ± 4.82		
Master’s degree and above	66 (15.00)	20.33 ± 5.11		
Monthly night shifts ^c^				
0	134 (30.45)	19.04 ± 4.51	6.904	0.001 ^e^
1–5	132 (30.00)	19.72 ± 5.02		
>5	174 (39.55)	21.03 ± 4.81		
Working experience ^c^				
≤10	126 (28.64)	21.31 ± 4.97	6.702	0.001 ^f^
11–20	178 (40.45)	19.30 ± 4.87		
>20	136 (30.91)	19.80 ± 4.49		
Employment type ^a^				
Permanent	366 (83.18)	19.81 ± 4.79	−2.101	0.036
Nonpermanent	74 (16.82)	21.11 ± 5.04		
Profession ^a^				
Psychiatrist	123 (27.95)	19.96 ± 4.65	0.195	0.845
Nurse	317 (72.05)	20.06 ± 4.93		

SD, standard deviation; ^a^ Student’s *t*-test; ^b^ Welch’s *t*-test; ^c^ one-way ANOVA; Bonferroni-corrected post hoc test: ^d^ Intermediate vs. Junior and below (*p* = 0.031); ^e^ >5 vs. 0 (*p* = 0.001); ^f^ 11–20 vs. ≤10 (*p* = 0.001); >20 vs. ≤10 (*p* = 0.034).

**Table 2:** Correlations among emotional exhaustion, emotional labor strategies, and quiet quitting.

Variables	Mean ± SD	Emotional Exhaustion	Surface Acting	Natural Expression	Deep Acting	Quiet Quitting
Emotional exhaustion	12.32 ± 3.93	1				
Surface acting	16.19 ± 5.41	0.538 *	1			
Natural expression	10.72 ± 2.51	−0.273 *	−0.437 *	1		
Deep acting	12.13 ± 3.19	−0.024	0.085	0.457 *	1	
Quiet quitting	20.03 ± 4.85	0.421 *	0.458 *	−0.407 *	−0.212 *	1

* *p* < 0.01.

**Table 3:** Hierarchical multiple linear regression.

Variables	Step 1				Step 2				Step 3			
	β	SE	t	*p*	β	SE	t	*p*	β	SE	t	*p*
Gender (female vs. male)	−0.169	0.589	−3.649	<0.001	−0.142	0.538	−3.372	0.001	−0.117	0.503	−2.973	0.003
Monthly night shifts (>5 vs. 0)	0.183	0.458	3.959	<0.001	0.172	0.418	4.085	<0.001	0.114	0.393	2.878	0.004
Working experience (11–20 vs. ≤10)	−0.140	0.456	−3.02	0.003	−0.107	0.417	−2.540	0.011	−0.083	0.387	−2.13	0.034
Emotional exhaustion					0.401	0.052	9.491	<0.001	0.223	0.057	4.807	<0.001
Surface acting									0.277	0.047	5.265	<0.001
Natural expression									−0.107	0.102	−2.039	0.042
Deep acting									−0.174	0.072	−3.706	<0.001
F	11.558				32.960				33.022			
*p*	<0.001				<0.001				<0.001			
R^2^	0.074				0.233				0.349			

**Table 4:** Mediation analysis of emotional labor strategies in the association between emotional exhaustion and quiet quitting.

Pathway	Effect Type	B	β	SE	t	*p*	LLCI	ULCI
Emotional exhaustion → Quiet quitting	Total effect	0.494	0.400	0.052	9.447	<0.001	0.391	0.597
Emotional exhaustion → Quiet quitting	Direct effect	0.282	0.228	0.058	4.820	<0.001	0.167	0.397
Emotional exhaustion → Surface acting	Direct effect	0.739	0.537	0.055	13.433	<0.001	0.631	0.847
Surface acting → Quiet quitting	Direct effect	0.195	0.217	0.046	4.285	<0.001	0.106	0.284
Emotional exhaustion → Natural expression	Direct effect	−0.166	−0.261	0.029	−5.732	<0.001	−0.223	−0.109
Natural expression → Quiet quitting	Direct effect	−0.413	−0.213	0.086	−4.782	<0.001	−0.582	−0.243
Emotional exhaustion → Surface acting → Quiet quitting	Indirect effect	0.144	0.116	0.037			0.071	0.217
Emotional exhaustion → Natural expression → Quiet quitting	Indirect effect	0.069	0.056	0.022			0.029	0.115

B, unstandardized coefficient; β, standardized coefficient; SE, standard error; LLCI, lower limit of the 95% confidence interval; ULCI, upper limit of the 95% confidence interval. LLCI and ULCI are based on unstandardized coefficients. Indirect effects are reported as unstandardized bootstrap estimates.

## Data Availability

The data supporting the findings of this study are available from the corresponding author upon reasonable request. The data are not publicly available due to ethical and privacy restrictions.

## Abbreviations

The following abbreviations are used in this manuscript:

HCWs	Healthcare workers
COR	Conservation of resources
ANOVA	One-way analysis of variance
CI	Confidence interval
EI	Expected influence
BEI	Bridge expected influence
CS	Correlation stability
SD	Standard deviation
SE	Standard error
EE	Emotional exhaustion
SA	Surface acting
NE	Natural expression
DA	Deep acting
WD	Work detachment
LI	Lack of initiative
LM	Lack of motivation
